# A national evaluation of the Irish public health counselling in primary care service– examination of initial effectiveness data

**DOI:** 10.1186/s12888-021-03226-x

**Published:** 2021-05-03

**Authors:** Charles Brand, Fiona Ward, Niamh MacDonagh, Sharon Cunningham, Ladislav Timulak

**Affiliations:** 1 School of Psychology, Trinity College, Dublin 2, Ireland; 2 Health Service Executive, Counselling in Primary Care National Evaluation, 19 Upper Ormond Quay, Dublin 2, Ireland; 3 Health Service Executive, 34 Brew’s Hill, Navan, Co, Meath, Ireland; 4 Health Service Executive, 1st Floor Junction House, Primary Care Centre, Airton Rd., Tallagh, Co, Dublin, Ireland; 5 Health Service Executive, Unit 8A Brulington Business Park, Srah Avenue, Tullamore, Co, Offaly, Ireland

**Keywords:** Psychotherapy research, Primary care counselling, Practice-based evidence, National counselling service evaluation, Logistic regression, Multi-level modelling

## Abstract

**Background:**

The Counselling in Primary Care service (CIPC) is the first and only nationally available public counselling service in the Republic of Ireland. This study provides initial data for the effectiveness of short-term psychotherapy delivered in a primary care setting in Ireland for the first time.

**Method:**

A practice-based observational research approach was employed to examine outcome data from 2806 clients receiving therapy from 130 therapists spread over 150 primary care locations throughout Ireland. Pre-post outcomes were assessed using the CORE-OM and reliable and clinically significant change proportions. Binary logistic regression examined the effect of pre therapy symptom severity on the log odds of recovering. Six and 12 month follow up data from a subsample of 276 clients were also analysed using growth curve analysis.

**Results:**

Of 14,156 referred clients, 5356 presented for assessment and 52.3% (*N* = 2806) consented to participate. Between assessment and post-therapy a large reduction in severity of symptoms was observed- Cohen’s *d* = 0.98. Furthermore, 47% of clients achieved recovery,a further 15.5% reliably improved, 2.7% reliably deteriorated and34.7% showed no reliable improvement. Higher initial severity was associated with less chance of recovering at post-therapy. Significant gains were maintained between assessment and12 months after therapy- Cohen’s *d* = 0.50.

**Conclusions:**

Outcomes for clients in the CIPC service compared favourably with large scale counselling and psychotherapy services in jurisdictions in the U.K., the U.S.A., Norway and Sweden. This study expands the international primary care psychotherapy research base to include the entire Republic of Ireland jurisdiction.

## Background

By 2001, in line with other national health agencies internationally, the Irish government had acknowledged the significant body of international evidence for the benefits of an integrated approach to the provision of psychological therapies within the context of primary care. The mental health policy framework document at the time entitled Vision for Change [1], highlighted the role of psychological therapies in addressing mental health difficulties stating they “...should be regarded as fundamental to basic mental health services.” [1]. However, while the Irish general public perceived psychological therapy services positively, they also reported high levels of dissatisfaction with the lack of availability, access and choice of services and a predominance of medication-only approaches to the treatment of mental health problems [2, 3].

Findings from a number of small scale studies in various parts of Ireland demonstrated that counselling was heavily utilised by GPs and effective in the areas where it was made available [4–6]. This evidence along with Irish Government mental health policy designed to increase provision of psychological therapies in primary care led to the establishment by the Health Service Executive (HSE) (Ireland’s national public health service provider) of the Counselling in Primary Care (CIPC) service in 2013. The CIPC service provides time-limited counselling contracts of up to 8 counselling sessions. We use the term counselling interchangeably with the word psychotherapy or psychological therapy as this terminology represents the Irish context and this specific service. CIPC practitioners must hold an accredited qualification in counselling or psychotherapy or counselling/clinical psychology. The Irish healthcare system is primarily funded through taxation, with contributions also provided by out-of-pocket payments and private health insurance. All adults in receipt of a public medical services card (provided to those whose weekly income is below a certain threshold) are eligible for provision of certain health care services free of charge, including counselling/psychotherapy as provided by CIPC. Further, within the Irish context of care, CIPC is aimed at those experiencing problems of mild to moderate severity, with help provided by a counsellor/therapist on a one to one basis. This is distinct from secondary care where presenting problems are of a more severe nature and clients are handled by multidisciplinary teams.

Practice-based studies provide empirical evidence for the effectiveness of counselling and psychotherapy in primary care in the treatment of depression and anxiety related problemsin routine practice [7, 8]. Other studies have been conducted in parts of the United Kingdom [9], Sweden [10], Norway [11] and the United States [12]. These evaluations add to the evidence base demonstrating that psychological therapies delivered in routine practice are effective in reducing levels of psychological distress.

Until this study, there were no national data regarding the effectiveness of counselling provided in the primary care context in the Republic of Ireland. We aimed to assess the initial available data which focussed on a typical cohort of CIPC clients, i.e. heterogeneous in the types of presenting problems, aged between the of 18 and 80, had attended the service for at least one therapy session and for whom complete pre and post counselling data were available (i.e. a Form-returner sample). We also sought to examine whether the effectiveness of counselling was impacted in the longer term by the initial severity of symptoms, as this is an important consideration for the delivery of the service.

Hence, this study was concerned with the following questions: (a) is psychological therapy provided by the national CIPC service clinically effective in reducing psychological distress for clients who complete a counselling episode in the short term; (b) what is the effect of the initial severity of participants’ symptoms on clients’chance of achieving recovery; and (c) are the gains achieved post-counselling retained between the end of counselling and at six and 12 months after counselling has ended?

## Method

### Procedure

We used a practice-based observational design in this study. Following a feasibility assessment in one service area in 2015, data collection for this study began in June 2016. Pre-post counselling data were collected across the remaining nine services between June 2016 and August 2019. A national centralised client administration system (CORENET™) was rolled out to the CIPC service in 2017 and outcome data for six of the nine areas were entered into this system as part of routine service delivery.^1^

All clients who presented for assessment were eligible for participation in the study. Counsellors completed the CORE Therapy Assessment Form at the beginning of Counselling and the End of Therapy Form when counselling concluded. The Clinical Outcome in Routine Evaluation Outcome Measurement Form (CORE-OM) was administered to all clients during their initial assessment session and again during the final session when attended [13–15]. The CORE-OM questionnaire was the primary outcome measure for this study that sought to assess the effectiveness of the CIPC service in relation to other services also utilising the CORE-OM, or similar measures of psychological distress.

All participants in the study were invited to provide follow-up outcome data six and 12 months after the end of their counselling. These questionnaires were posted directly to clients in accordance with their counsellingend date and to their last known address with a return stamped addressed envelope in order for them to return completed questionnaires directly to their respective CIPC area coordination offices. Follow up data were inputted into the CORENET system by HSE area administrators.

### Participants

The national average annual number of referrals to the CIPC service over the last 3 years was approximately 17,000^2^ clients. The total number of referrals recorded during this study was 14,156. Data collection in three areas was for three months only, as opposed to 12 months in the other six areas. In order to compare the number of clients in the service for the period of the study with previous years, the number of participants in areas with shorter collection periods were adjusted to reflect a 12 month data collection period and added to the total number of referrals in the remaining areas. This gave an estimated total number of referrals of 17,486, broadly in line with the current average annual referral rate to the service.

During this study, 37.6% of referrals did not progress to the assessment stage because referees did not complete the mandatory opt-in process. A further 24.6% of referrals were considered inappropriate for reasons including referees being under the age of 18; not in possession of a valid public services card or due to the nature or severity of their problem. Overall, 5356 clients were assessed during the course of the study and of these 84.1% were accepted for therapy and invited to participatein the study (*n* = 4505) and of these 2806 (62.3%) consented to take part in the study. After an initial screening by area coordinators the suitability of all patients referred into CIPC to undergo counselling is completed by Counsellor/Therapists on a client-by-client basis. Reasons why assessments end for reasons other than ‘Accepted for therapy’ can include ‘Referred to another service’, ‘Unsuitable for therapy at this time’ and ‘Accepted for trial period of therapy’. Counsellor/Therapists may also describe other reasons why particular clients are not suitable for therapy at that time.

**Fig. 1:**
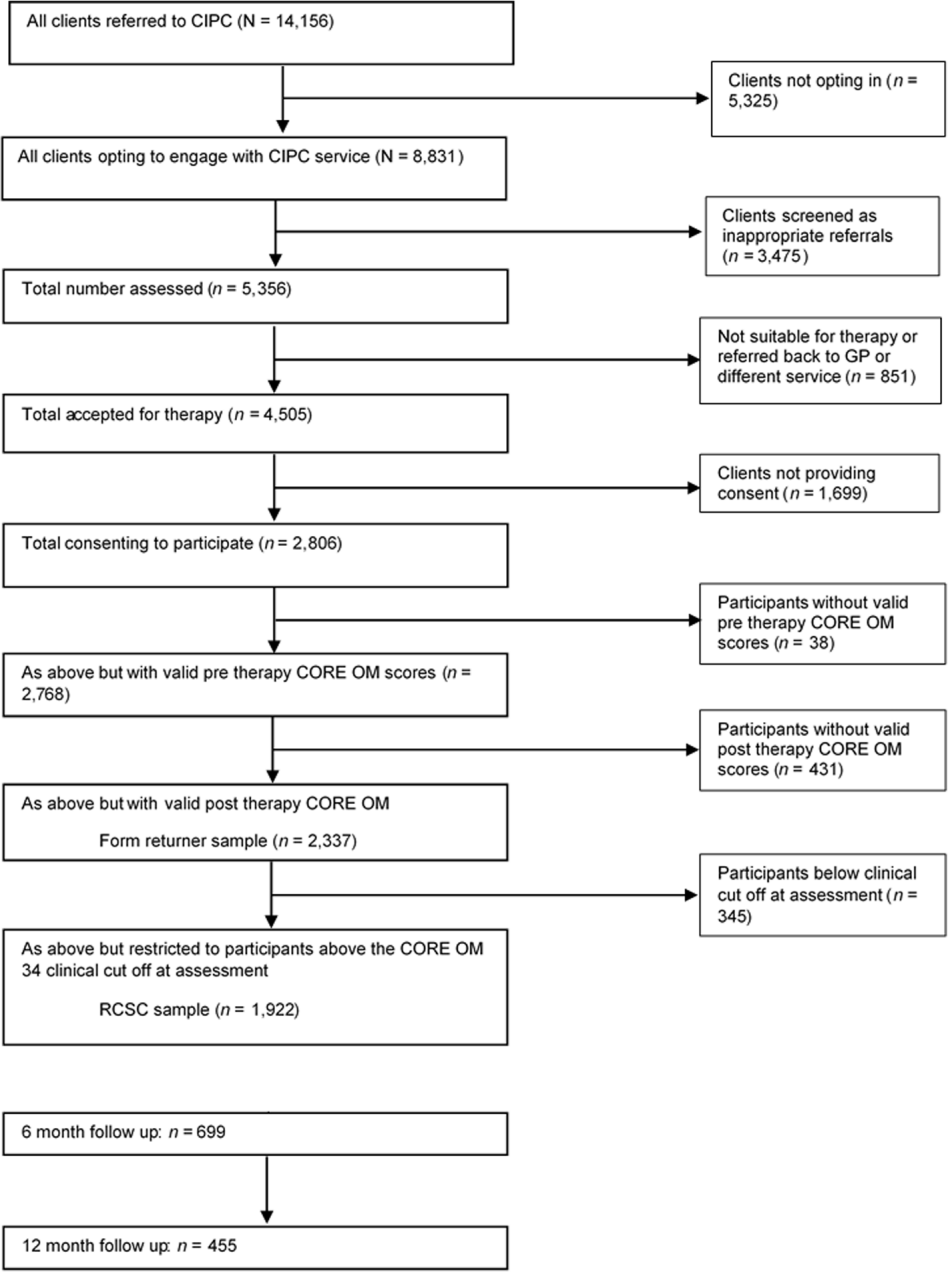
CONSORT diagram of participation in the study

Of the total number of 2806 clients whom consented to participate in the study (*n* = 2806), 98.6% (*n* = 2768) completed pre-therapy CORE-OM and 83.3% (*n* = 2337) completed post-therapy questionnaires (to comprise the Form-returner sample). A total of 24.9% (*n* = 699) returned six month follow up questionnaires and 16.2% (*n* = 455) returned 12 month follow up CORE-OM questionnaires (see Fig. 1). The average attendance for the Form-returner sample was 7.97sessions (*SD* = 3.57) and 82.1% were above the clinical threshold of 1.00 on the CORE-OM.

### Measures

#### The CORE-OM

The primary outcome measure used in the study was the CORE-OM [14, 16]. This is a 34 item global measure of distress and is used extensively in clinical settings across primary and secondary services in Ireland, the UK and Europe. The CORE-OM comprises 34 items addressing domains of subjective well-being (4 items), symptoms (12 items), functioning (12 items) and risk (6 items: 4 ‘risk to self’ items and 2 ‘risk to others’ items). The total mean score is calculated by dividing the total score by the number of completed item responses. The cut-off point to delineate between clinical and non-clinical populations in this study was set at a CORE-OM score of 1.00, which is used in many studies of this type using the same measure [9, 18–20].

#### Reliable and clinically significant change (RCSC)

A reliable change index (RCI) was calculated for each participant in the Form-returner sample. The RCI is a pre-post difference summarising change for each participant in the context of changes in the overall sample [21] and is equal to the individual’s score before counselling, minus their score after counselling and divided by the standard error of the difference of the test (see [22] for formulae). The 1-month test–retest reliability of .88 as reported by Barkham et al. [23] was used along with the standard error of the pre–post difference (*SD*_diff_) of .30 which was calculated from all participants with valid pre and post-counsellingCORE-OM scores – i.e. the Form-returner sample. This resulted in an RCI of .53 which was used as the reference amount to determine whether pre-post differences in participants’ CORE scores could have occurred by chance alone.

### Data analysis

#### Pre-post analysis

A repeated measure t-test was conducted to determine whether there were significant differences in participants’ pre and post-counselling scores on the CORE-OM. Cohen’s *d* effect size was also calculated for participants in the Form-returner sample meeting criteria for inclusion in the pre-post analyses described above (*n =* 2337). A client was deemed recovered if their pre-counselling CORE-OM score had moved from being above the clinical range (equal to or greater than 1.00), to the non-clinical range and reliably improved (i.e. that is by a margin of at least 0.53). Pre-post outcomes in terms of reliable change (improvement) were also calculated, i.e., when the pre-post score decreased by more than 0.53. Clients whose pre-post score was less than 0.53 (in either direction) were considered to be showing ‘no reliable change’ and those whose score increased by more than 0.53 were considered ‘deteriorated’. For the RCSC analyses, only clients with valid pre and post CORE-OM scores and who scored above the predetermined cut-off point on the CORE-OM measure at assessment (*n* = 1922) were included in calculations determining recovery, because it is not possible for participants who score below the cut-off point at assessment to move from above to below the cut-off point and hence be considered recovered.

#### Pre-therapy CORE-OM scores as a predictor of outcome

Using recovery (i.e. recovered/not recovered) as a binary dependent variable and participants’ pre-counselling scores on the CORE-OM as a measure of severity and the independent variable, a logistic regression analysis was conducted to determine whether pre–counselling CORE-OM scores predicted participants’ log odds of recovery achievement.

### Long term outcomes

A growth curve model was tested to investigate whether there was non-linear change in CORE-OM scores over time once counselling ended and 12 months later. In the analytical approach adopted to examine longitudinal change, time was used as an explanatory variable at level 1, which essentially defines it as a longitudinal model [24]. Hence, participants’ change trajectories in CORE-OM scores over time were examined (a within-subjects factor with three levels – post- counselling, six months later and at 12 months after counselling) for the purpose of determining if post-counselling changes on the CORE-OM were maintained over the longer term and if there were differences in the rate of change between participants.

There were large numbers of non-returned questionnaires from eligible participants (*n* = 2806) for follow up analyse sat six and 12 month follow up time points - 73% and 84.6% respectively. When employing growth curve analyses, high proportions of missing data at level 2 (in this case at the participant level) in a two level design such as the one used here, can introduce unacceptable levels of bias in the resulting parameter estimates [25, 26]. While methods for the imputation of missing data are available e.g. multiple imputation and full maximum likelihood estimation (see 25,and 26 for further information), the proportions of missing data in this case were considerable. Hence, a list-wise deletion approach was adopted. This meant that only those participants with valid CORE-OM questionnaires at all three measurements points for the period of interest were included in the analyses i.e. post-counselling, six month follow up and 12 month follow up (*n* = 276). These participants displayed slightly higher of CORE-OM scores, (*t* = − 2.82, df = 2766, *p* = .005, one-tailed).

### Analytical approach

**Fig. 2:**
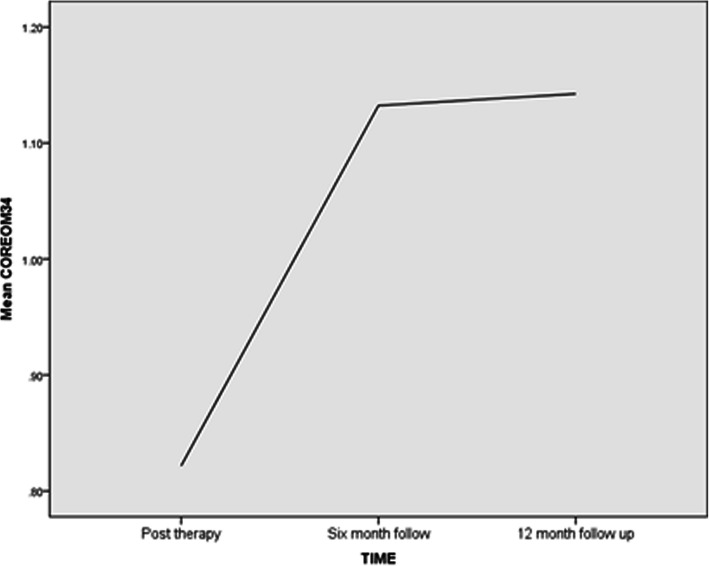
Line graph showing non-linear growth between post counselling, six month follow up and 12 month follow up (*n* = 276)

The default time variable was centred such that the intercept corresponded to the initial time point (i.e. the post-counselling CORE-OM measurement) to make changes in the time components easier to interpret once included in the model. Descriptive statistics showed considerable variation in post-counselling CORE-OM scores (range 0.03 to 3.30). Further, certain characteristics of the data were ascertained before embarking on the specification and generation of the multi-level models. A visual inspection plotting the mean change trajectory suggested a non-linear shape to the aggregate changes in CORE-OM scores over the three time points (see Fig. 2). Hence, both linear and quadratic variables were added to subsequent models in order to better describe the data. The time variable was recoded into a squared quadratic sequence (0, 1,and 4) and this term was added to the model as a fixed effect to capture any changes in the rate of change that might occur over three measurement time points. A Maximum Likelihood (ML) estimation method was used in order to facilitate the comparison of the resulting models [25].

First, a model without time related variables (Model A) was generated in order to investigate whether a sufficient amount of variation existed between participants’ CORE-OM scores after counselling to warrant further investigation into the contribution of time to existing variation.

The combined equation for Model A:

$$ COREOM{34}_{ti}={\beta}_{00}+{u}_{0i}+{\varepsilon}_{ti} $$

This was considered a baseline intercept only model, which does not include explanatory variables (i.e. it was unconditional) and estimated three parameters: the fixed effect describing average CORE-OM score, between participant random variance and Level one residual variance.

Next, in order to capture rates of change over time both linear and quadratic terms were added to the overall model (Model B). Each participant’s successive measurement over time was defined by their growth trajectories and random measurement error at Level 1. Differences in the trajectories of groups of participants at Level 2 could then be examined.

The combined equation for Model B:

$$ COREOM{34}_{ti}={\beta}_{00}+{ ti me}_{ti}+{\beta}_{20}{Quadtime}_{ti}+{u}_{1i}{ ti me}_{ti}+{u}_{0i}+{\varepsilon}_{ti}. $$

## Results

### Descriptive statistics

Females accounted for 74.8% (*n* = 2099) and the mean age of participants was 42.6 years (*Mdn* = 41, *SD* = 14.57). Waiting times for initial assessments were available for 93.5% (*n* = 2265) and showed a mean time of 4.53 months (*Mdn* = 4.0, *SD* = 2.35). For 12% of the sample (*n* = 338) ethnic origin data were either not available or missing and of the remaining 88% of participants,73.4% (*n* = 2059) were recorded as White Irish and 11.3% (*n* = 318) as Any Other White Background with each of the remaining ethnic categories accounting for less than 1% individually.

Some differences were observed when the overall sample was split by participants for whom pre and post therapy CORE-OM scores were available and those for whom only post therapy scores are recorded. Participants for whom only pre-therapy data were available were slightly younger (40.8 (SD14.4)) than older participants (42.8 (SD14.5)) (*t* = − 2.6, df = 2716, *p* = 0.009) and waited longer for therapy to begin (5.3 months (SD2.6)) as opposed to (4.4 (SD2.2)) (*t* = 7.3, df = 2587, *p* < 0.001). No differences were observed in terms of proportions above the CORE-OM cut-off point at pre-therapy, sex, employment, ethnic origin or being a previous client of the CIPC service.

The rate of missing data in the overall sample (*n* = 2806) for the CORE-OM measure at baseline was 1.3%. Rates of missing data for other variables included in the analysis were: Waiting time in weeks 6.5%, Type of ending to counselling episode 4.1%, Number of sessions attended 5.0%, Employment status 8.4%. Follow up rates for post counselling CORE-OM scores at post counselling was 16.6%, at six month follow up 73% and 12 month follow up 84.6%.

### Pre-post change

There was a significant decrease in global distress between pre counselling and post-counselling as indicated by an overall reduction in mean CORE-OMscores from 1.57 to 0.95 (*t* = 47.057, df = 2337, *p* = <.0001, one-tailed). This resulted in an effect size of Cohen’s *d* = 0.98 (see Table 1).

**Table 1:** Pre to post therapy form returner sample CORE OM scores, differences and effect sizes

		Pre therapy		Post therapy						95% CI	
Measure	*N*	*M*	*SD*	*M*	*SD*	*t*	df	*p*	*d*	*LL*	*UL*
CORE OM	2337	1.57	0.605	0.95	0.654	47.057	2336	<.0001	0.98	0.594	0.645

*Note.* Form returner sample = All clients with valid pre and valid post therapy therapy CORE OM scores, attended at least 1 therapy session, *M* Mean, Cohen’s d calculation = Cohen’s *d* = (M2 - M1) / SDpooled, LL = Lower Limit, UL = Upper Limit

### Reliable and clinically significant change (improvement and recovery rates)

Of the overall sample of 2337 clients with pre and post data, 82.2% (*n* = 1922) scored above the CORE-OM clinical cut-off point (i.e. 1.00) at pre-counselling assessment stage. Post-counselling, 46.9% (*n* = 902) met criteria for recovery (reliable and clinically significant change). When clients who showed statistically significant improvement only (15.5%, *n* = 298) are included an overall improvement rate of 62.4% was found. Of the remaining participants, 2.7% (*n* = 52) reliably deteriorated while 34.7% (*n* = 666) showed no reliable improvement (see Table 2).

**Table 2:** Reliable and clinically significant change, reliable improvement, no relaible change and reliable deterioration proportions for participants in form returner sample above clinical threshold (*n* = 1922)

		RCSC		Reliable improvement		Reliable deterioration		No reliable change	
Sample	*N*	*n*	%	*n*	%	*n*	%	*n*	%
Form returner sample	1922	902	46.9	298	15.5	52	2.7	666	34.7

*Note.* Form returner sample = valid pre and post therapy therapy CORE OM scores, pre therapy CORE OM 34 scores > = 1.00, attended at least 1 therapy session

### Initial severity as a predictor of recovery

The results of the independent t-test showed pre counselling CORE-OM scores as being significantly different between the group achieving and not achieving recovery (*U* = 345,137.500, *N*_1_ = 854, *N*_2_ = 974, *p* < .001). A logistic regression was performed with pre counselling CORE-OM scores added a as predictor variable. A total of 1922 cases were analysed. The full model significantly predicted recovery status (omnibus chi-square = 42.480,df = 1, *p* = <.001). The model accounted for between 2.2 and 2.9%of the variance in recovery status, with 67% of those non-recovered and 46% of participants who recovered successfully predicted, giving an overall accurate prediction rate of 57%. The β coefficient(− 0.637) and Wald statistic (41.166, *p* < .001) indicated that participants with higher initial CORE-OM scores were less likely to end counselling as recovered. For every additional one unit increase in pre counselling CORE-OM scores, the log odds of recovery decreased by a factor of 0.53 (95% CIs: [0.44–0.64]).These results indicate that initial severity was an indicator of outcome whereby those clients starting counselling with higher levels of overall distress were associated with less chance of recovering.

### Long-term results

As reported earlier, there were significant improvements in symptomatology between pre and post-counselling – a mean reduction of .62 on the CORE-OM. However, a question remained about the behaviour of outcomes between post-counselling and 12 month follow up in terms of the rate and significance of that change, a question addressed in the following growth curve analysis.

Results from Model A (i.e. null model) showed a significant grand mean CORE-OM score across all participants *β*_00_ = 1.02, *p* < .001. The level 1 residual variance estimate, which summarises the difference between each participant’s observed and predicted CORE-OM score over time was significant (0.207, *p* < .001). There was also significant variation in the estimate describing variation in the intercepts across participants at level 2(0.245, *p* < .001) indicating significant differences in CORE-OM scores between participants over time. An intra class coefficient (ICC) was calculated to estimate the proportion of the variation in CORE-OM scores (see 25 for calculation details). This estimate provided evidence of substantial clustering between participants grouped at different time points, where 54.2%of the variation in CORE-OM scores occurred at Level 1.

Next linear and quadratic terms were added to the null model to capture rates of change over time and the slopes were allowed to vary over participants at level 2 (but the quadratic term was fixed). The parameter estimate for the intercept was *β*_00_ = 0.81, *p* < .001 and the time variable had been rescaled as described earlier. This estimate now reflected the average CORE-OM score for participants at the first time measure, i.e. post counselling. The linear time component estimate *β*_10_ = .42, *p* < .001 indicated a significant increase in the CORE-OM grand mean score (i.e. 0.81) of 0.42.The quadratic term describing was also significant (*β*_20_ = − .13, *p* < .001) suggesting the growth curve was non-linear and both the linear and quadratic terms were contributing to the overall model. The quadratic growth component estimated together with the linear component suggested that, on average, the rate of increase in CORE-OM scores slowed between post-counselling and 12 month follow up (see Table 3).

**Table 3:** Growth curve models estimating the association between CORE OM scores and total number of sessions attended at post therapy, six month and 12 month follow up (*N* = 276)

Fixed effects	Model A	Model B
Intercept *β*_00_	1.02 (.03)**	0.81 (.04) **
*time β*_10_		.42 (.07)**
*quadtime β*_20_		−.13 (.03)**
Random effects		
Residual *ε*_*ti*_	.207 (.01)**	.14 (.02)**
Intercept *π*_2*i*_	.245 (.02)**	.21 (.03)**
Covariance *u*_0*i*_		.001 (.006)*
Slope *π*_1*i*_		.008 (.003)**
−2 Log Likelihood	1462.521	1357.640
Number of estimated parameters	3	7

*Note:* Parameter estimate standard errors listed in parentheses

** *p* < .001

*p = .859

The Level-1 residual (*ε*_*ti*_ =.14, *p* < .01) suggested significant variance in observed versus predicted CORE-OMscores within participants and significant variation across participants at post-counselling (*π*_2*i*_ =.21, *p* < .001). The slope variance estimate also indicated significant variance in the CORE-OM growth trajectories between participants (π_1*i*_ = 0.008, *p* = .002). However, the covariance between the intercept and slope did not vary significantly from zero (*u*_0*i*_ = 0.006, *p* = .859).

## Discussion

The Irish health service (HSE) established a national counselling service (CIPC) in July 2013 for adults experiencing mild to moderate psychological difficulties. We sought to provide the international counselling/psychotherapy research community, mental health policy makers, potential service users and other stakeholders with effectiveness data for Ireland’s national primary care counselling service for the first time.

This study found a significant positive effect of counselling and a reduction in global distress for participants attending CIPC. Our observed recovery rate of 46.9% is broadly in line with that reported by a similar service - 50.8% reported by the UK’s primary care Increasing Access to Psychological Therapy Service (IAPT) [27]. Our effect size and recovery rate findings (Cohen’s *d* = 0.98 and 46.9% respectively) are also in line with those reported in other large-scale practice-based studies of primary care counselling and psychotherapy services in the UK [9, 20], Sweden [10] and Norway [11] where effect sizes between 0.51 and 1.47 and recovery rates between 40.3 and 65% were observed. The use of a common measurement system (i.e. the CORE-OM), makes for easier comparisons between services in different jurisdictions providing a clearer comparative assessment of effectiveness. The large Form-returner sample in this study, combined with the low threshold for participant inclusion in the pre post analysis, allows for a high degree of generalisability of the outcome results to both the population of users of primary care counselling services in Ireland and similar client groups using comparable services in other countries.

Logistic regression results indicated that participants with higher initial CORE-OM scores were less likely to achieve recovery, a finding in line with previous similar approaches to addressing this question [28]. CORE-OM benchmarks for services comparable with CIPC have shown that average rates of recovery for non-severe clients are appreciably higher than for clients who are more severely distressed [29]. Our results in terms of severity of symptoms at the onset of counselling and how this relates to recovery also align with those reported in IAPT (e.g. 33). In a study of outcomes and treatment duration in UK routine practice, Stiles, Barkham and Wheeler [18] identified greater severity at intake and fewer treatment gains for secondary and tertiary care clients. They related this difference to problem complexity, which they suggest responds to treatment more slowly. Previous studies have found that the application of additional counselling sessions does not, in itself necessarily equate directly with clinically improved outcomes in a linear manner at the aggregate level [30]. Rather, the number of sessions attended by each client is related to the rate at which their symptoms change [31, 32], with less severe clients taking less sessions to improve. A “one size fits all” approach to deciding treatment length is not supported by research, but should be decided on a case-by-case basis [32]. This would suggest that our study warrants further investigation in relation to the CIPCtime-limited model of service utilised with clients’ who present with greater severity at assessment.

A growth curve model was developed to determine whether there was a non-linear change in global distress over time between the end of counselling and 12 months later. The results indicated that for participants in the study for whom measurements were available between the end of counselling and 12 months later there was a significant linear increase in CORE-OM scores. However, the rate of increase significantly slowed over time. Growth curve analysis estimated the overall average CORE-OM score of clients in this study at post-counselling was 0.81. This increased by an average 0.42 over the following 12 months. Importantly however, gains demonstrated between post-counselling and 12 months later stayed significantly below pre-counselling levels. The rate at which the increase slowed was significantly different among participants, but these differences were small. Similar investigations into the longer term effects of counselling and psychotherapy have found broadly similar results. Ray-Sannerud et al. [33] reported a similar pattern of recovery in clients attending clinics in the Primary Health Care model of service in the U.S.. In response, the potential benefits of providing counselling “top-up” sessions could be considered for suitable CIPC clients. Long term gains in behavioural change have been associated with follow up counselling to encourage physical activity [34] and Wu et al. [35] also found single follow up counselling sessions decreased the likelihood of relapse in smoking prevention programs.

## Limitations

As in most practice-based studies examining therapy effectiveness, this study was not designed as a controlled randomised experiment and therefore lacks the comparative mechanism of atypical RCT, i.e. a control group. However, the primary purpose of this study was less about addressing questions of causality or the comparative performance of therapy approaches than establishing a national baseline for the immediate and longer term effectiveness of counselling in Ireland, with the additional objective of comparing the outcomes with comparable services elsewhere.

Attrition at follow up was a feature in this study as is the case in many practice-based studies especially when conducting research with a primary care population and on a national scale [36]. The current study also experienced difficulties obtaining follow up data. Importantly however, while participants in this study exhibited slightly higher CORE-OM scores at follow up than at pre counselling, this difference was small in clinical terms. An additional limitation was a loss of data over the follow up period. Some data were lost due to administrative changes over the course of the study associated with the adoption of a new client data tracking system during which time follow up data were incorrectly entered into the system.

Researchers conducting practice-based studies acknowledge the inevitable attrition which occurs between pre and post therapy during normal service operations, and have posited reasons for such occurrences, e.g. readiness of clients to end counselling without further contact with the service because they feel their therapy has been successful or do not feel they are receiving the type of help they need, or because external factors (i.e. transport issues, lack of support network) mean they can no longer attend [10]. However, for practice based studies which seek to examine the longer affects of counselling such as this one, such sizable numbers of participants for whom data are not returned can pose a significant threat to the internal validity of the long term analysis of outcomes. While not within the scope of this study, methods of imputing missing data for hierarchically structured data for use in growth curve analyses can be incorporated into longitudinal outcome analysis (see [37, 38]).

Drop out between referral and assessment in this study was 62.2% (*n* = 8800) which is roughly in line with other similar services. For example, the IAPT service in the UK reported a 57.7% drop between referral and assessment [39]. The CIPC assessment process resulted in 851 clients being referred back to their GP or onto another service leaving 4505 clients accepted for counselling. However, 37.7% of clients assessed for counselling did not agree to participate in the study. Possible factors include a reluctance on the part of some therapists to invite clients to take part in the research or client reservations about participation [40].

Pre post analyses in this study focussed on the Form-returner sample only (only participants with valid pre and post questionnaire data). Some previous practice-based studies examining outcomes in large services have used pre- counselling outcome scores as post-counselling scores when this data is missing (e.g. 9), this inevitably results in more conservative outcome results.

## Conclusions

In line with other large-scale studies, this study found significant improvement in self-reported distress levels for the majority of clients attending short-term counselling in Ireland. While over the course of 12 months after counselling the effect of counselling diminished, it did not to do so by a statistically significant amount. Those with more severe problems before attending counselling were less likely to significantly improve.

This study is significant as it provides the first national effectiveness data for a counselling service deployed at national level in Ireland and suggests positive outcomes for the majority of service users once counselling ends, with some evidence of longer lasting effects. Further research on identifying clients that could benefit from additional “top-up” counselling sessions, and the effect those sessions might have on longer term effectiveness is warranted. The results from this study are relevant to and can inform services in other jurisdictions as well as psychotherapy researchers internationally, mental health policy makers in Ireland and key stakeholders in the CIPC service.

## Data Availability

The datasets used and/or analysed during the current study are available from the corresponding author on reasonable request.

## Abbreviations

CIPC: Counselling in Primary Care
CORE-OM: Clinical Outcome in Routine Evaluation Outcome Measurement
HSE: Health Service Executive
IAPT: Increasing Access to Psychological Therapy
RCI: Reliable Change Index
RCSC: Reliable and Clinically Significant Change
RCT: Randomised Control Trial

## Footnotes

1 The other three areas mentioned recorded client data and responses using pen and paper.

2 This average is for 9 of the ten CIPC Community Hospital Organisations (CHOs). One CHO area was not part of this phase of the research

## References

[1] Department of Health (2006). Vision for Change : Report of the Expert Group on Mental Health Policy [Internet].

[2] Batt V, Nic Gabhainn S, Falvey F (2002). Perspectives on the provision of counselling for women in Ireland. Women’s Studies Centre and Centre for Health Promotion Studies, National University of Ireland, Galway.

[3] HSRC NCS (2003). SENCS : survivors’ experiences of the National Counselling Service. For adults who experienced childhood abuse.

[4] Bourke M, Byrne M (2012). Evaluation of a pilot primary care adult mental health practitioner – delivered service. Ir Psychol.

[5] Martin E, Hawkins S, Hicks T, O’Flynn M. Psychological services for adults in primary care. Ir Psychol. 2008; [cited 2014 Jul 2]; Available from: http://www.lenus.ie/hse/handle/10147/271913.

[6] Ward F, McGrath T. To a life that shines ten years transforming the shadows [Internet]. Dublin: Health Service Executive (HSE); 2010. p. 252. Available from: http://hdl.handle.net/10147/112142.

[7] Ammerman A, Smith TW, Calancie L (2014). Practice-based evidence in public health: improving reach, relevance, and results. Annu Rev Public Health.

[8] Barkham M, Hardy GE, Mellor-Clark J (2010). Developing and delivering practice-based evidence [internet].

[9] Barkham M, Stiles WB, Connell J, Mellor-Clark J (2012). Psychological treatment outcomes in routine NHS services: What do we mean by treatment effectiveness?. Psychol Psychother Theory Res Pract.

[10] Werbart A, Levin L, Andersson H, Sandell R (2013). Everyday evidence: outcomes of psychotherapies in Swedish public health services. Psychotherapy..

[11] Knapstad M, Nordgreen T, ORF S. Prompt mental health care, the Norwegian version of IAPT: clinical outcomes and predictors of change in a multicenter cohort study. BMC Psychiatry. 2018;18 [cited 2019 Aug 25]. Available from: https://www.ncbi.nlm.nih.gov/pmc/articles/PMC6097447/. 10.1186/s12888-018-1838-0 PMC6097447 30115041

[12] Sawchuk CN, Craner JR, Berg SL, Smyth K, Mack J, Glader M, Burke L, Haggerty S, Johnson M, Miller S, Sedivy S, Morcomb D, Heredia D, Williams MW, Katzelnick DJ (2018). Initial outcomes of a real-world multi-site primary care psychotherapy program. Gen Hosp Psychiatry.

[13] Barkham M (2005). Suitability and utility of the CORE-OM and CORE-A for assessing severity of presenting problems in psychological therapy services based in primary and secondary care settings. Br J Psychiatry.

[14] Mellor-Clark J (2006). Developing CORE performance indicators for benchmarking in NHS primary care psychological therapy and counselling services: an editorial introduction. Couns Psychother Res.

[15] Mellor-Clark J, Barkham M, Connell J, Evans C (1999). Practice-based evidence and standardized evaluation: informing the design of the CORE system. Eur J Psychother Couns.

[16] CORE System Group (1998). CORE System (Information Management) Handbook [Internet]. Leeds: CORE System Group.

[17] Connell J, Barkham M, Stiles WB, Twigg E, Singleton N, Evans O, Miles JNV (2007). Distribution of CORE-OM scores in a general population, clinical cut-off points and comparison with the CIS-R. Br J Psychiatry.

[18] Stiles WB, Barkham M, Wheeler S (2015). Duration of psychological therapy: relation to recovery and improvement rates in UK routine practice. Br J Psychiatry.

[19] Skre I, Friborg O, Elgarøy S, Evans C, Myklebust LH, Lillevoll K (2013). The factor structure and psychometric properties of the clinical outcomes in routine evaluation – outcome measure (CORE-OM) in Norwegian clinical and non-clinical samples. BMC Psychiatry.

[20] Stiles WB, Barkham M, Mellor-Clark J, Connell J (2008). Effectiveness of cognitive-behavioural, person-centred, and psychodynamic therapies in UK primary-care routine practice: replication in a larger sample. Psychol Med.

[21] Evans C, Margison F, Barkham M (1998). The contribution of reliable and clinically significant change methods to evidence-based mental health. Evid Based Ment Health.

[22] Jacobson NS, Truax P (1991). Clinical significance: a statistical approach to defining meaningful change in psychotherapy research. J Consult Clin Psychol.

[23] Barkham M, Mullin T, Leach C, Stiles WB, Lucock M (2007). Stability of the CORE-OM and the BDI-I prior to therapy: evidence from routine practice. Psychol Psychother Theory Res Pract.

[24] Raudenbush SW, Bryk AS (2002). Hierarchical linear models: applications and data analysis methods.

[25] Heck RH, Thomas SL, Tabata LN, 2nd edition (2014). Multilevel and longitudinal modeling with IBM SPSS.

[26] Peugh JL (2010). A practical guide to multilevel modeling. J Sch Psychol.

[27] NHS Digital (2018). Psychological Therapies: Annual Report on the use of IAPT services, England, further analyses on 2016–17 [Internet]. The Health and Social Care Information Centre.

[28] Saxon D, Ivey C, Young T (2008). Can CORE assessment data identify those clients less likely to benefit from brief counselling in primary care?. Couns Psychother Res.

[29] Mullin T, Barkham M, Mothersole G, Bewick BM, Kinder A (2006). Recovery and improvement benchmarks for counselling and the psychological therapies in routine primary care. Couns Psychother Res.

[30] Barkham M, Rees A, Stiles WB, Shapiro DA, Hardy GE, Reynolds S (1996). Dose–effect relations in time-limited psychotherapy for depression. J Consult Clin Psychol.

[31] Barkham M, Connell J, Stiles WB, Miles NV, Margison F, Evans C (2006). Dose-effect relations and responsive regulation of treatment duration: The good enough level. J Consult Clin Psychol.

[32] Falkenström F, Josefsson A, Berggren T, Holmqvist R (2016). How much therapy is enough? Comparing dose-effect and good-enough models in two different settings. Psychotherapy..

[33] Ray-Sannerud BN, Dolan DC, Morrow CE, Corso KA, Kanzler KE, Corso ML, Bryan CJ (2012). Longitudinal outcomes after brief behavioral health intervention in an integrated primary care clinic. Fam Syst Health.

[34] Gagliardi AR, Faulkner G, Ciliska D, Hicks A (2015). Factors contributing to the effectiveness of physical activity counselling in primary care: a realist systematic review. Patient Educ Couns.

[35] Wu L, He Y, Jiang B, Zuo F, Liu Q, Zhang L, Zhou C (2016). Additional follow-up telephone counselling and initial smoking relapse: a longitudinal, controlled study. BMJ Open.

[36] Saxon D, Ricketts T, Heywood J (2010). Who drops-out? Do measures of risk to self and to others predict unplanned endings in primary care counselling?. Couns Psychother Res.

[37] Sterne JAC, White IR, Carlin JB, Spratt M, Royston P, Kenward MG, et al. Multiple imputation for missing data in epidemiological and clinical research: potential and pitfalls. BMJ. 2009;338(jun29 1):b2393. 10.1136/bmj.b2393 PMC2714692 19564179

[38] Chakraborty H, Gu H. A mixed model approach for intent-to-treat analysis in longitudinal clinical trials with missing values. Research Triangle Park (NC): RTI Press; 2009. [cited 2019 Nov 7]. (RTI Press Methods Report Series). Available from: http://www.ncbi.nlm.nih.gov/books/NBK538904/30896910

[39] Gyani A, Shafran R, Layard R, Clark DM. Enhancing recovery rates: lessons from year one of IAPT. Behav Res Ther. 2013;51(9):597–606. 10.1016/j.brat.2013.06.004. 10.1016/j.brat.2013.06.004 PMC3776229 23872702

[40] Draper H, Wilson S, Flanagan S, Ives J (2009). Offering payments, reimbursement and incentives to patients and family doctors to encourage participation in research. Fam Pract.

