# Correction to: A national evaluation of the Irish public health counselling in primary care service– examination of initial effectiveness data

**DOI:** 10.1186/s12888-021-03286-z

**Published:** 2021-06-01

**Authors:** Charles Brand, Fiona Ward, Niamh MacDonagh, Sharon Cunningham, Ladislav Timulak

**Affiliations:** 1 School of Psychology, Trinity College, Dublin 2, Ireland; 2 Health Service Executive, Counselling in Primary Care National Evaluation, 19 Upper Ormond Quay, Dublin 2, Ireland; 3 Health Service Executive, 34 Brew’s Hill, Navan, Co, Meath, Ireland; 4 Health Service Executive, Primary Care Centre, 1st Floor Junction House, Airton Rd., Tallagh, Co, Dublin, Ireland; 5 Health Service Executive, Unit 8A Brulington Business Park, Srah Avenue, Tullamore, Co, Offaly, Ireland

**Correction to: BMC Psychiatry 21, 227 (2021)**

**https://doi.org/10.1186/s12888-021-03226-x**

Following the publication of the original article [1], the authors identified an error in the affiliation of Dr. Ladislav Timulak.

The correct affiliation is given below:

1 School of Psychology, Trinity College, Dublin 2, Ireland.
